# Profile of Gaze Dysfunction following Cerebrovascular Accident

**DOI:** 10.1155/2013/264604

**Published:** 2013-10-10

**Authors:** Fiona J. Rowe, David Wright, Darren Brand, Carole Jackson, Shirley Harrison, Tallat Maan, Claire Scott, Linda Vogwell, Sarah Peel, Nicola Akerman, Caroline Dodridge, Claire Howard, Tracey Shipman, Una Sperring, Sonia MacDiarmid, Cicely Freeman

**Affiliations:** ^1^Department of Health Services Research, Thompson Yates Building, University of Liverpool, Brownlow Hill, Liverpool L69 3GB, UK; ^2^Altnagelvin Hospitals HHS Trust, Altnagelvin BT47 6SB, UK; ^3^NHS Ayrshire and Arran, Ayr KA6 6DX, UK; ^4^Royal United Hospitals Bath NHS Trust, Bath BA1 3NG, UK; ^5^Bury Primary Care Trust, Bury BL9 7TD, UK; ^6^Durham and Darlington Hospitals NHS Foundation Trust, Durham DH1 5TW, UK; ^7^Ipswich Hospital NHS Trust, Ipswich IP4 5PD, UK; ^8^Gloucestershire Hospitals NHS Foundation Trust, Gloucester GL1 3NN, UK; ^9^St Helier General Hospital, Jersey JE2 3QS, UK; ^10^University Hospital NHS Trust, Nottingham NG7 2UH, UK; ^11^Oxford Radcliffe Hospitals NHS Trust, Oxford OX3 9DU, UK; ^12^Salford Primary Care Trust, Salford M6 8HD, UK; ^13^Sheffield Teaching Hospitals NHS Foundation Trust, Sheffield S10 2TB, UK; ^14^Swindon and Marlborough NHS Trust, Swindon SN3 6BB, UK; ^15^Wrightington, Wigan and Leigh NHS Trust, Wigan WN1 2NN, UK; ^16^Worcestershire Acute Hospitals NHS Trust, Worcester WR5 1DD, UK

## Abstract

*Aim*. To evaluate the profile of ocular gaze abnormalities occurring following stroke. *Methods*. Prospective multicentre cohort trial. Standardised referral and investigation protocol including assessment of visual acuity, ocular alignment and motility, visual field, and visual perception. *Results*. 915 patients recruited: mean age 69.18 years (SD 14.19). 498 patients (54%) were diagnosed with ocular motility abnormalities. 207 patients had gaze abnormalities including impaired gaze holding (46), complete gaze palsy (23), horizontal gaze palsy (16), vertical gaze palsy (17), Parinaud's syndrome (8), INO (20), one and half syndrome (3), saccadic palsy (28), and smooth pursuit palsy (46). These were isolated impairments in 50% of cases and in association with other ocular abnormalities in 50% including impaired convergence, nystagmus, and lid or pupil abnormalities. Areas of brain stroke were frequently the cerebellum, brainstem, and diencephalic areas. Strokes causing gaze dysfunction also involved cortical areas including occipital, parietal, and temporal lobes. Symptoms of diplopia and blurred vision were present in 35%. 37 patients were discharged, 29 referred, and 141 offered review appointments. 107 reviewed patients showed full recovery (4%), partial improvement (66%), and static gaze dysfunction (30%). *Conclusions*. Gaze dysfunction is common following stroke. Approximately one-third of patients complain of visual symptoms, two thirds show some improvement in ocular motility.

## 1. Introduction

Ocular motility (eye movement) problems are reported commonly following stroke in up to 68% of cases [1–5]. These problems can include cranial nerve palsy [6], vergence and accommodative dysfunction [3], strabismus [2, 7], and nystagmus [8]. Such eye movement abnormalities can cause symptoms of diplopia, blurred vision, compensatory head posture, nausea, and dizziness because of the inability to move one or both eyes into a particular gaze direction [1, 9, 10]. These symptoms can impact on activities of daily living and quality of life by impairing reading ability and hindering mobility because of instability [11].

Gaze abnormalities may include horizontal and/or vertical conjugate gaze palsy, internuclear ophthalmoplegia (INO), one and a half syndrome, and saccadic and smooth pursuit palsy [12]. Much of the medical literature describing these gaze abnormalities is in the form of case reports and small case series of individual types of gaze abnormality [13–17]. However, there are few large scale studies documenting these problems in stroke populations. We sought in this paper to evaluate the profile of ocular gaze abnormalities following stroke in a large, prospective, observation study of stroke survivors with visual impairment.

## 2. Methods and Materials

The design of this study was a prospective multicentre observational case cohort study. The Vision In Stroke (VIS) group consists of local investigators from twenty UK hospital trusts who are responsible for assessing stroke patients and collecting patient data. The data is collated centrally at the University of Liverpool. The study has multicentre ethical approval via the National Research Ethics Service and is being undertaken in accordance with the Tenets of Helsinki. The recruitment period for this study ran from May 2006 to April 2009.

The target population was stroke patients suspected of having a visual difficulty. Referrals could be made from in-patient wards, rehabilitation units, community services, or outpatient clinics. Patients were given an information sheet and recruited after providing informed, written consent. Patients were excluded if they were unable to consent due to cognitive impairment or unwilling to consent, if their diagnosis was that of transient ischaemic attack or other neurological pathologies, or if they were discharged without vision assessment.

Patients with suspected visual difficulty were identified using a screening form which became the referral form to the orthoptic service. Suspected visual difficulty was based on the presence of patient-reported visual symptoms or examiner-observed signs of visual impairment. A standardised investigation sheet was used for the eye assessment consisting of identification of known preexistent ocular pathology, symptoms, and signs and investigation of visual field, ocular motility, and perceptual aspects. Visual acuity was assessed at near and distance fixation with Snellen or logMAR acuity tests. Assessment of ocular alignment and motility consisted of cover test, evaluation of saccadic, smooth pursuit and vergence eye movements, retinal correspondence (Bagolini glasses), fusional vergence (20D or fusional range), stereopsis (Frisby near test), prism cover test, and lid and pupil function. Complete loss of ocular rotation was defined as a palsy and partial loss of ocular rotation was defined as paresis. Perceptual deficits were recorded after questioning the patient and/or carers and relatives. Inattention was assessed by means of a combination of assessments including line bisection, Albert's test, cancellation tests, and memory tests using verbal description and drawing. Stroke details were recorded from patient notes (from CT or MRI reports) accounting for stroke laterality, type, and area involved. Ocular treatment details were recorded along with outcome.

Results were inputted to the statistical package SPSS version 19 (IBM SPSS Statistics, USA). Pearson chi-squared test was undertaken to analyse cross-tabulations of results for gaze palsy versus factors such as location and laterality of stroke, associated ocular and nonocular signs, and symptoms and outcome. Nonparametric assessment (Wilcoxon test) was undertaken to compare categorical data.

## 3. Results

### 3.1. General Demographics

1345 patients were referred for visual assessment for this study. 915 patients were recruited and 430 patients were excluded, the latter mainly due to inability to consent. Of the 915 patients recruited, 59% were males and 41% females. Mean age at onset of stroke was 69.18 years (range 1–94: SD 14.19 years). Median duration from onset of stroke to initial eye examination was 22 days (0–2543 days), with the mean of 40.84 (SD 141.28) days being skewed by three outliers (patients referred a number of years after the stroke onset). Stroke lesion was right sided in 48.9% (i.e., right sided brain damage), left sided in 37.7%, and bilateral in 13.4%. Infarcts accounted for 84.5% including thrombosis and embolism. Haemorrhagic strokes accounted for the remainder.

### 3.2. Ocular Motility Abnormalities

498 patients (54%) were diagnosed with ocular motility abnormalities. Of these, 207 (41.5% of ocular motility abnormalities and 23% of overall cohort) patients had gaze abnormalities whether isolated or combined with other ocular deficits. Gaze abnormalities included impaired gaze holding (*n* = 46), complete gaze palsy/paresis (*n* = 23), horizontal gaze palsy/paresis (*n* = 16), vertical gaze palsy/paresis involving both upward and downward gaze (*n* = 17), Parinaud's dorsal midbrain syndrome (*n* = 8), INO (*n* = 20), one and half syndrome (*n* = 3), saccadic palsy/paresis (*n* = 28), and smooth pursuit palsy/paresis (*n* = 46). 

### 3.3. Location of Stroke

**Figure 1:**
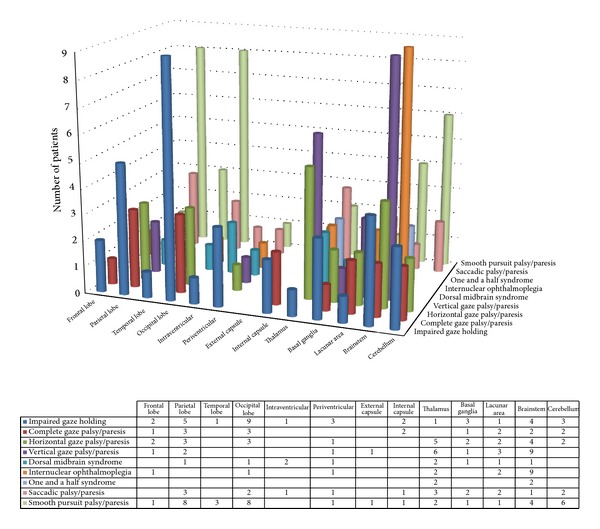
Area of stroke lesion.

Of the 207 patients with gaze abnormalities, 84% of strokes were infarctions and the remainder were due to haemorrhage. The areas of brain affected by the stroke were the cerebellum, brainstem, and diencephalic areas (inclusive of thalamus and basal ganglia) in 46% of stroke lesion locations (Figure 1). In addition, strokes causing gaze dysfunction involved cortical areas including occipital, parietal, and temporal lobes, intra- and periventricular areas, and external and internal capsule plus lacunar strokes, accounting for 54%. Smooth pursuit, saccadic, and gaze holding deficits were seen more frequently with occipital, parietal, and temporal lobe strokes. INO and one and a half syndrome were more frequently seen with brain stem and diencephalic strokes. There was no significant difference between specific types of gaze abnormalities caused by infarctions or haemorrhages.

### 3.4. Symptoms and Ocular Alignment

Visual symptoms were reported in 58.5% (*n* = 121) inclusive of diplopia, blurred vision, visual field loss, reading difficulty, and oscillopsia (Table 1). Diplopia was the most common visual symptom in these cases. Manifest strabismus was documented in 37% (*n* = 77) including exotropia, esotropia, hypertropia, and hypotropia, with exotropia occurring most commonly (Table 2). Many patients (42%) with manifest strabismus did not complain of diplopia. 

### 3.5. Associated Ocular Abnormalities

**Figure 2:**
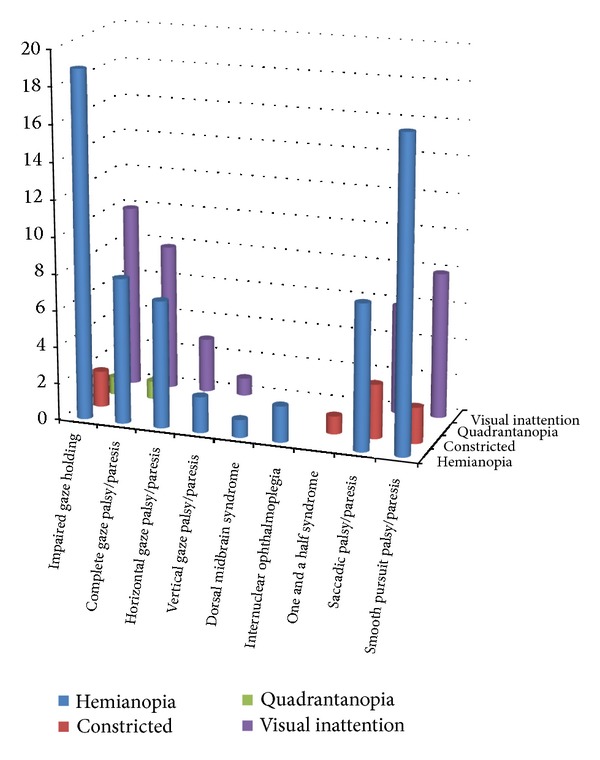
Associated visual field loss and inattention.

Gaze abnormalities occurred as isolated ocular motility impairments in 104 cases (50%) and in association with other ocular motility and visual abnormalities in 103 cases which included impaired convergence, nystagmus, and lid or pupil abnormalities (Table 3). Furthermore, visual field loss was present in 36% (*n* = 74), of which 46% reported symptoms of visual loss. Visual inattention was noted in 17% (*n* = 36) as outlined in Figure 2. Visual field loss and visual inattention were more frequently associated with smooth pursuit, saccadic, and impaired gaze holding reflecting cortical strokes of the occipital, parietal, and temporal lobes.

### 3.6. Rehabilitation and Outcome

A variety of management strategies were provided for 77% of patients (Table 4). Advice was provided in 33% which consisted of targeting adaptive strategies to aid visual symptoms including the use of compensatory head posture, visual field awareness, visual scanning, and reading options and the use of appropriate task lighting to optimise visual function. Prisms and/or occlusion were prescribed as the most common intervention and were predominantly required to alleviate diplopia. 

37 patients were discharged after initial orthoptic assessment and treatment for diagnoses of impaired gaze holding (*n* = 13), Parinaud's syndrome (*n* = 1), gaze palsy (*n* = 5), INO (*n* = 2), saccadic paresis (*n* = 2), smooth pursuit paresis (*n* = 11), horizontal gaze paresis (*n* = 2) and vertical gaze paresis (*n* = 1). 29 patients were referred to other ophthalmic services after initial orthoptic assessment and treatment for impaired gaze holding (*n* = 11), Parinaud's syndrome (*n* = 1), gaze palsy (*n* = 4), INO (*n* = 2), saccadic paresis (*n* = 5), smooth pursuit paresis (*n* = 3), horizontal gaze paresis (*n* = 2), and vertical gaze paresis (*n* = 1). 141 were offered review appointments for diagnoses of impaired gaze holding (*n* = 23), Parinaud's syndrome (*n* = 6), gaze palsy (*n* = 13), one and a half syndrome (*n* = 3), INO (*n* = 16), saccadic paresis (*n* = 21), smooth pursuit paresis (*n* = 32), horizontal gaze paresis (*n* = 12) and vertical gaze paresis (*n* = 15). Of the patients offered review appointments, 28 did not attend their follow-up appointment and six patients died before follow-up. 107 patients attended for review (Table 5) showing full recovery (4%), partial improvement (66%), and static gaze dysfunction (30%). Patients showing full recovery had diagnoses of impaired gaze holding (*n* = 2) and smooth pursuit paresis (*n* = 2). There was no significant difference in types of gaze abnormalities for patients who showed partial improvement versus those with no recovery. Improvement was noted over periods of 2 weeks to 6 months. Those with static gaze dysfunction showed no change from their baseline assessment through to follow-up assessments. 

## 4. Discussion

The VIS study involves a subpopulation of stroke, that is, those already suspected of having a visual problem. Consequently, our results are not reflective of the general stroke population. Of all patients referred with suspected visual impairment, 54% had ocular motility abnormalities and of these 41.5% were due to gaze abnormalities (these accounted for 23% of all recruited patients). Gaze abnormalities are a common occurrence following brain injury [3]. Karatas [18] has reported that supranuclear disorders, which include gaze abnormalities, account for approximately 10% of all patients with disorders of eye movements. The gaze abnormalities documented in our study included impaired gaze holding, complete gaze palsy/paresis, horizontal gaze palsy/paresis, vertical gaze palsy/paresis, dorsal midbrain syndrome, INO, one and a half syndrome, saccadic (fast eye movements) palsy/paresis, and smooth pursuit (slow eye movements) palsy/paresis. 

Half of the gaze abnormalities in our study occurred as isolated visual impairment and the remaining half were associated with additional ocular motility abnormalities and visual problems such as cranial nerve palsy, saccadic dysmetria, nystagmus, impaired convergence, and lid and pupil anomalies plus visual field loss and visual inattention. The ability to make full ocular rotations combined with neural integration to maintain eye position is required for normal gaze holding. Thus, gaze holding can be impeded by cortical stroke involving eye movement pathways or causing visual field loss or brainstem/cerebellum strokes involving structures that mediate gaze such as the interstitial nucleus of Cajal, nucleus prepositus hypoglossi, and medial vestibular nuclei [12, 19, 20]. Furthermore, impaired gaze holding may occur in the recovery process of gaze palsies. Our cases of impaired gaze holding were due to occipital/parietal lobe stroke as well as strokes in brainstem and cerebellar areas. 

Complete gaze palsy in which there are impaired horizontal and vertical eye movements is often seen in conditions such as Parkinson's disease and progressive supranuclear palsy [21] but has been reported following stroke. Lee and colleagues [22] reported a case of total horizontal gaze palsy with concurrent loss of vertical saccades and pursuits due to a small dorsal caudal pontine infarct. Cases have also been reported of bilateral ophthalmoplegia due to bilateral paramedian midbrain-thalamic infarction [23]. Horizontal gaze palsy involved loss of eye movement to the right, left, or both sides. Such deficit is typically seen following pontine lesions [15, 17, 24] where there is involvement of the sixth nerve nucleus from which horizontal gaze for both saccadic and smooth pursuit movement is mediated [19, 20]. Dependent on the extent of infarction, other ocular motility abnormalities may be noted such as nystagmus, as was evident in our cases. Combined horizontal gaze palsy and INO is termed one and a half syndrome, and this was present in three cases with typical associated nystagmus and defective horizontal gaze on lateral eye movements. 

INO was documented in a further 20 patients, many of whom with impaired convergence which is an indicator of a rostral midbrain lesion. However, INO may be due to infarction at many levels of the brainstem including caudal and rostral pons, isthmus, and midbrain [25] and is due to interference with interconnections between the sixth nerve nucleus and contralateral medial rectus subnucleus via the medial longitudinal fasciculus [19, 20]. All our cases were unilateral as is usual for INO caused by infarction [26]. 

Vertical gaze palsy/paresis involved both upward and downward eye movements and was frequently associated with nystagmus and lid retraction or ptosis. The sites of stroke were often midbrain and diencephalic structures. Vertical gaze is generated from third and fourth nerve output with bilateral integration generated via interconnections of nerve fibres through the posterior commissure and brachium conjunctivum. Projections also involve the thalamus, interstitial nucleus of Cajal, and rostral interstitial nucleus of the medial longitudinal fasciculus [19, 20]. Reports in the literature often show infarction in the areas of the thalamus [16, 27] and midbrain [14, 28]. We also documented cases of dorsal midbrain syndrome, also known as Parinaud's syndrome, which is specifically a limitation of upgaze of both eyes and may show additional characteristics of convergence retraction nystagmus and light-near pupil dissociation. This occurs following involvement of vertical upward gaze projections through the posterior commissure in the upper midbrain [19, 20].

Saccadic and smooth pursuit eye movement palsy/paresis were common. Smooth pursuits can be impaired following parietal/occipital lobe lesions but also by brainstem and cerebellum lesions. Saccadic palsy/paresis can be due to stroke affecting many cortical and brainstem areas. A range of stroke lesions were documented in this study giving rise to saccadic and smooth pursuit deficits, which was not surprising given the extensive pathways for control of these eye movements [12, 19, 20, 29]. 

Acquired manifest strabismus was noted in 37% of our cases. Misalignment of the visual axes is expected where the gaze pattern of both eyes is asymmetrical and/or in which one is limited in its movement more than the other. Manifest strabismus is expected most for gaze abnormalities such as INO and one and a half syndrome. However, it may be seen in gaze palsies where the misalignment is attributable to coexistent ocular motility abnormalities such as cranial nerve palsy or cortical strabismus [6]. Vertical deviations of hypertropia or hypotropia were recorded, some of which were due to cranial nerve palsy but others due to skew deviation. Exotropia was most commonly detected and this is similar to previous reports of strabismus in stroke populations [2, 6]. 

Visual field loss was noted in 36% and 17% had visual inattention. It was not surprising to discover visual field loss associated with saccadic and smooth pursuit problems and impaired gaze holding as visual search eye movements are well reported as abnormal to the side of the visual field loss [30, 31]. Combined cortical and brainstem strokes typically accounted for the associated visual field loss with ocular motility conditions of horizontal/vertical gaze palsy/paresis and INO. 

Visual symptoms were reported in 58.5% of our population of gaze abnormalities with the most common symptom being diplopia due to the presence of manifest strabismus. Diplopia was not reported by all patients with manifest strabismus although some patients reported blurred vision rather than frank diplopia. This finding has been previously reported [2, 6]. A proportion of patients with visual field loss were symptomatically aware of their loss of vision. Other reported symptoms were oscillopsia, blurred vision, and reading difficulties. 

Khan and colleagues [32] stated that most visual impairments can be addressed by simple, yet effective, treatment options. Furthermore, treatment of patients with oculomotor based symptoms has been reported as successful in 90% of patients with sustained improvement in their symptoms [33]. Treatment was targeted at individual symptoms in our study. Frequently, prisms and occlusion were utilised for relief of diplopia, refraction was undertaken for those complaining of blurred vision, and advice was provided to aid adaptation to reading difficulty and visual field loss. 52% of patients attended the follow-up review appointments of which 70% showed partial or full recovery and importantly no deterioration of ocular motility. 

For those with little or no improvement in gaze deficit, it is important to consider further management options. Compensatory head postures can be used to adapt to small areas of diplopia on side gaze in addition to compensating for impaired gaze to one side. Furthermore, yoked prisms may be used to shift images towards a central position where there is an inability to move gaze in one direction. Prisms and/or occlusion may also be utilised monocularly for the symptom of diplopia and jumbled vision. For long-standing symptoms of diplopia and large head postures, ocular motility surgery or botulinum toxin may be required and the required procedures are determined by the type and extent of gaze abnormality [34–37]. Altered spectacle correction may be warranted for those with blurred vision, and it is important to consider the type of spectacles in patients with unresolved vertical gaze palsy. Bifocals and varifocals can prove problematic particularly when downgaze is so limited that the patient has significant impairment with many activities of daily living such as reading, walking, and going downstairs; in these cases, single focus lenses are usually the most effective option for rehabilitation and prevention of falls. Early intervention with targeted strategies to move the head, scan the environment, or utilise prisms, alongside appropriate refractive correction, can speed up rehabilitation and support early discharge. Certification of visual impairment registration can also be offered to individuals whose restricted eye movements cause impact on activities of daily living.

The impact of ocular motility abnormalities and related symptoms is such that there is a detrimental effect on activities of daily living and quality of life [11]. Currently one in six persons is aged 65 years or older and by 2050 it is projected that this will be one in four [38]. An increase in the number of strokes may also occur concurrently with the ageing population leading to increased referrals of those with visual impairment. There is thus a likely impact on the NHS and provision of such services and visual rehabilitation options as outlined above. 

In this study, orthoptists evaluated ocular motility by assessment of ocular alignment, range and extent of eye movements, vergence, and binocular vision. Thus, an accurate determination of ocular motility was possible. It is difficult to obtain accurate assessment of ocular motility without such formal scrutiny and accuracy is imperative to detect subtle changes of eye movement that may cause significant impairment. Scales such as the National Institute for Health stroke scale (NIHSS) [39] record eye movement disorders, but, importantly, this is limited to partial gaze palsy and forced deviation. The scale evaluates the best gaze and horizontal eye movements only. Thus, vertical gaze disorders such as those found in this study would not be documented. Screening assessments based on questioning and observation have also been found to lack accuracy in comparison to detailed orthoptic evaluation [40]. Given the impact of eye movement abnormalities on activities of daily living and quality of life, it is important that accurate assessment is made and appropriate rehabilitation implemented. It has been recommended that vision rehabilitation specialists are an important part of the stroke multidisciplinary rehabilitation team [32]. 

There are a number of limitations to our study. We were unable to obtain detailed brain imaging reports for many cases and therefore had to reply on written reports in the patient case records. Thus, a detailed analysis of anatomical location of stroke versus type of gaze palsy could not be undertaken. Review data was available for approximately half of our patients. Those whose gaze abnormality resulted in little or no visual symptoms were discharged, a number of patients were referred to other ophthalmology services, and a further small number did not attend their follow-up. We were therefore unable to establish the full extent of recovery of gaze abnormalities for all cases. 

## 5. Conclusions

Gaze dysfunction is common following stroke occurring in 23% of stroke patients with suspected visual impairment and accounting for 41.5% of all ocular motility abnormalities. Stroke lesions were located in cortical, brainstem, and cerebellar areas. Half were isolated gaze abnormalities and the remaining half were associated with additional visual impairments. Approximately one-third of patients complained of visual symptoms and two-thirds show some improvement in ocular motility. However, one-third did not recover from gaze dysfunction.

## Figures and Tables

**Table 1:** Associated visual symptoms.

	Diplopia	Blurred vision	Reading difficulty	Visual field loss	Oscillopsia
Impaired gaze holding	4	6		7	10
Complete gaze palsy	5	2	1	6	
Horizontal gaze palsy	5	1		3	
Vertical gaze palsy	8	3		1	
Dorsal midbrain syndrome	2	3			
INO	12				
One and a half syndrome	2				
Saccadic palsy	2	4	1	9	
Smooth pursuit palsy	5	9	2	8	

**Table 2:** Ocular alignment.

	Exotropia	Esotropia	Eso-/hypotropia	Exo-/hypotropia	Hypertropia	Hypotropia
Impaired gaze holding	7	1		2		3
Complete gaze palsy	4	2	1			
Horizontal gaze palsy	2	2		1	3	
Vertical gaze palsy	4	1		1	5	
Dorsal midbrain syndrome	1	2		1		
INO	9			4		
One and a half syndrome		1			1	
Saccadic palsy	6				1	
Smooth pursuit palsy	7			1	4	

**Table 3:** Associated ocular motility abnormalities.

	CNP	Saccadic dysmetria	Impaired elevation	Impaired depression	Impaired SP	INO	Nystagmus	Impaired convergence	Lid anomaly	Pupil anomaly
Impaired gaze holding	1	5	4		2		End: 1 Gaze: 2 Jerk: 3 Multi: 1	17	Bilat: 1 Retract: 1 Unilat: 7	Anisoc: 1 Horners: 1 Mid: 1 Miosis: 1 RAPD: 1

Complete gaze palsy	3	3	2		1	1	Gaze: 5 Upbeat: 3	11	Retract: 1 Unilat: 4	Anisoc: 1 Horners: 1 Mid: 1

Horizontal gaze palsy	3	6	5	1	1		End: 1 Gaze: 2 Jerk: 2 Multi: 1 Rotary: 1 Upbeat: 2	4	Retract: 1 Unilat: 6	Anisoc: 2

Vertical gaze palsy	4	6			2	2	CRN: 2 Down: 1 Gaze: 1 Jerk: 2 Rotary: 1 Upbeat: 2	7	Retract: 2 Unilat: 8	Anisoc: 3 Mid: 1

Dorsal midbrain syndrome	1	2					CRN: 4 Gaze: 1	2	Retract: 1	LND: 2

INO		6	8		1		Abduct: 2 CRN: 1 End: 1 Gaze: 1 Upbeat: 2	13	Unilat: 6	Anisoc: 1 Delay: 1

One and a half syndrome							Abduct: 1 Jerk: 1	2	Unilat: 1	

Saccadic palsy			3		7	1	End: 3 Jerk: 1 Rotary: 1	11	Bilat: 1 Unilat: 5	Delay: 2 Mid: 2 Miosis: 1

Smooth pursuit palsy	3	20	9				CRN: 1 End: 3 Gaze: 1 Jerk: 2 Pendular: 1 Rotary: 4 Upbeat: 4	13	Bilat: 1 Unilat: 6	Colob: 1 Mid: 1 Miosis: 1

CNP: cranial nerve palsy; SP: smooth pursuit; INO: internuclear ophthalmoplegia; Abduct: abducting nystagmus; CRN: convergence retraction nystagmus; Down: downbeat nystagmus; End: end point nystagmus; Gaze: gaze evoked nystagmus; Jerk: horizontal jerk nystagmus; Multi: multivector nystagmus; Bilat: bilateral ptosis; Retract: lid retraction; Unilat: unilateral ptosis; Colob: iris coloboma; Delay: sluggish reaction; LND: light-near dissociation; Mid: mid-dilated pupils; RAPD: relative afferent pupillary defect.

**Table 4:** Rehabilitation options.

	Occlusion	Fresnel prisms	Refraction	Orthoptic exercises	Advice
Impaired gaze holding	2	4	8		18
Complete gaze palsy	3	3			10
Horizontal gaze palsy	2	6	1		5
Vertical gaze palsy	9	3			5
Dorsal midbrain syndrome	1	1	1		2
INO	12	2	1		2
One and a half syndrome	2				
Saccadic palsy	2	3	1		12
Smooth pursuit palsy	7	5	10	1	15

**Table 5:** Review outcome.

	Complete recovery	Partial recovery	Static	Died before follow-up
Impaired gaze holding	2	8		2
Complete gaze palsy		12	2	2
Horizontal gaze palsy		8	6	
Vertical gaze palsy		14	3	
Dorsal midbrain syndrome		4	4	
INO		6		
One and a half syndrome		2		
Saccadic palsy		6	11	1
Smooth pursuit palsy	2	11	6	1
